# Author Correction: Structure and mechanism of oxalate transporter OxlT in an oxalate-degrading bacterium in the gut microbiota

**DOI:** 10.1038/s41467-023-41872-9

**Published:** 2023-09-28

**Authors:** Titouan Jaunet-Lahary, Tatsuro Shimamura, Masahiro Hayashi, Norimichi Nomura, Kouta Hirasawa, Tetsuya Shimizu, Masao Yamashita, Naotaka Tsutsumi, Yuta Suehiro, Keiichi Kojima, Yuki Sudo, Takashi Tamura, Hiroko Iwanari, Takao Hamakubo, So Iwata, Kei-ichi Okazaki, Teruhisa Hirai, Atsuko Yamashita

**Affiliations:** 1 Research Center for Computational Science, Institute for Molecular Science, National Institutes of Natural Sciences, Okazaki, 444-8585 Japan; 2 Graduate School of Medicine, Kyoto University, Kyoto, 606-8501 Japan; 3 Graduate School of Medicine, Dentistry and Pharmaceutical Sciences, Okayama University, Okayama, 700-8530 Japan; 4 RIKEN SPring-8 Center, Sayo, 679-5148 Japan; 5 School of Pharmaceutical Sciences, Okayama University, Okayama, 700-8530 Japan; 6 Graduate School of Environmental and Life Sciences, Okayama University, Okayama, 700-8530 Japan; 7 Research Center for Advanced Science and Technology, The University of Tokyo, Tokyo, 153-8904 Japan

**Keywords:** X-ray crystallography, Computational biophysics, Bacterial structural biology, Membrane proteins

Correction to: *Nature Communications* 10.1038/s41467-023-36883-5, published online 03 April 2023

The original version of this Article contained an error in Fig. 2, in which “Y328A” was wrongly labelled as “W352A” in panel E, and in Fig. 3, in which residues “N42”, “N265” and “N268” were wrongly labelled as “Q42”, “Q265” and “Q268” in panel C. These have been corrected in both the PDF and HTML versions of the Article.

In the main text, in the first paragraph of the Results in the section “Ligand-free outward-facing structure”, “Gln264” was wrongly described as “Asn264” in both the PDF and HTML versions of the Article.

The original version of the Supplementary Information associated with this Article contained an error in Supplementary Fig. 5a, in which the legend for the plots was missing. The HTML has been updated to include a corrected version of the Supplementary Information.

### Supplementary information

Updated Supplementary Information

