# Supplementary material for: Structure and mechanism of oxalate transporter OxlT in an oxalate-degrading bacterium in the gut microbiota
Source: Nat Commun. 2023 Apr 3;14:1730. doi: 10.1038/s41467-023-36883-5 (PMC10070484; doi:10.1038/s41467-023-36883-5)
Supplement: Supplementary file 1 — Supplementary Information [file 41467_2023_36883_MOESM1_ESM.pdf]

Supplementary information

Structure and mechanism of oxalate transporter OxlT in an oxalate-degrading bacterium in the gut microbiota

Titouan Jaunet-Lahary, Tatsuro Shimamura*, Masahiro Hayashi, Norimichi Nomura, Kouta Hirasawa, Tetsuya Shimizu, Masao Yamashita, Naotaka Tsutsumi, Yuta Suehiro, Keiichi Kojima, Yuki Sudo, Takashi Tamura, Hiroko Iwanari, Takao Hamakubo, So Iwata, Kei-ichi Okazaki*, Teruhisa Hirai*, Atsuko Yamashita*

*To whom correspondence should be addressed:
Tatsuro Shimamura, t.shimamura@mfour.med.kyoto-u.ac.jp
Kei-ichi Okazaki, keokazaki@ims.ac.jp
Teruhisa Hirai, teruhisa.hirai@jst.go.jp
Atsuko Yamashita, a_yama@okayama-u.ac.jp

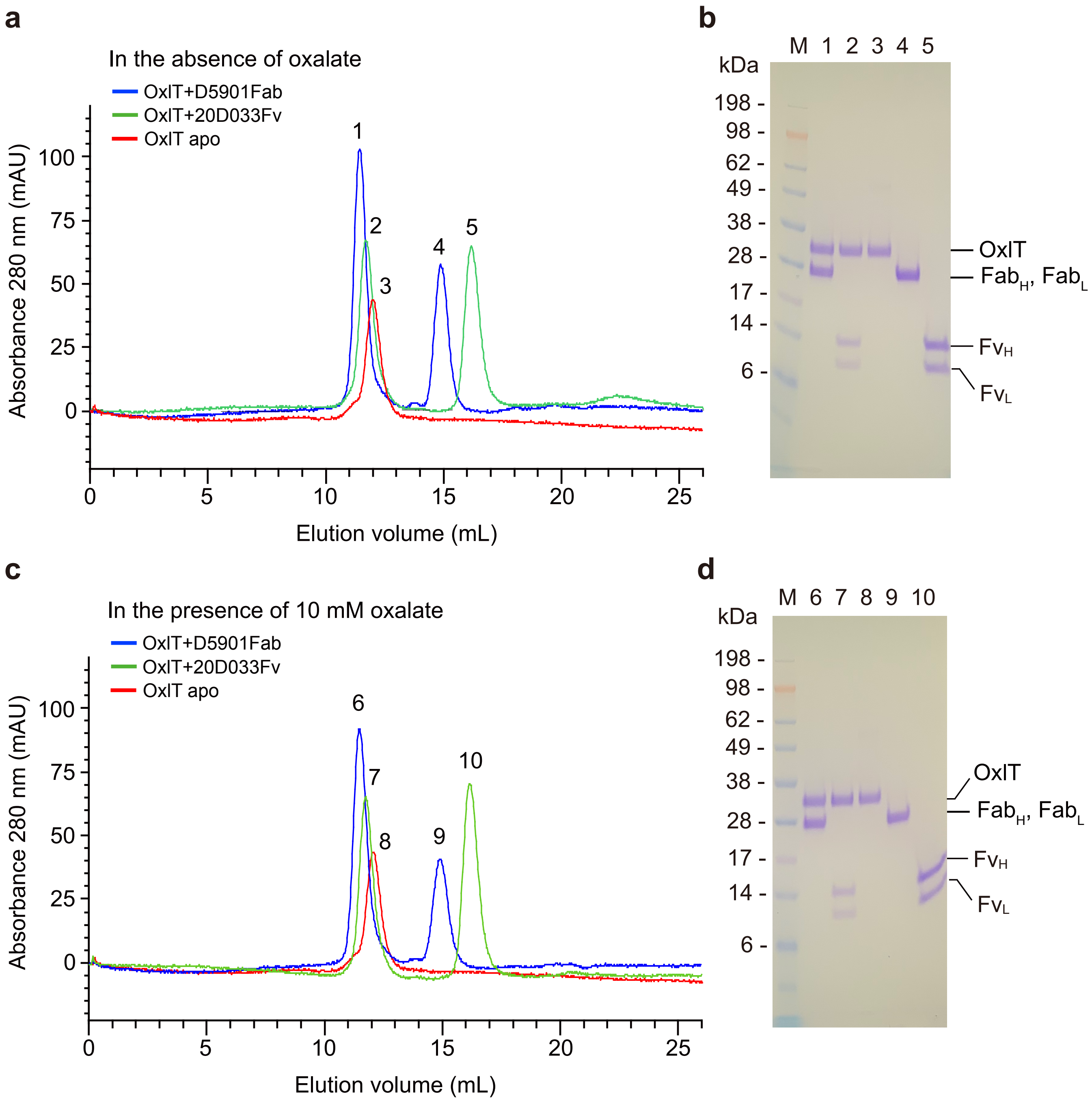

Supplementary Figure 1. **The Fab and Fv fragments used for crystallization bind to OxlT both in the presence and absence of oxalate. a** Typical size exclusion chromatography (SEC) profile of the OxlT-antibody complexes in the absence of oxalate (OxlT-Fab complex, blue line; OxlT-Fv complex, green line). A Superdex200 Increase 10/300GL column was equilibrated with a buffer composed of 20 mM MES-KOH (pH 6.2), 100 mM potassium acetate, and 0.02% DDM. OxlT (0.16 mg protein) was mixed with the excess antibody fragment (0.32 mg protein), incubated for 1 h on ice, and subjected to the SEC. OxlT alone (0.16 mg protein) was separated on the same column as a control (red line). Peak 1: OxlT-D5901Fab complex; peak 2: OxlT-20D033Fv complex; peak 3: free OxlT; peak 4: free D5901-Fab; and peak 5: free 20D033Fv. **b** SDS-PAGE analysis of the corresponding peak fractions in (a). Fab_H, Fab_L, Fv_H, and Fv_L represent the heavy and light chains of D5901Fab, as well as the heavy and light chains of 20D033 Fv fragments, respectively. **c** Typical size exclusion chromatography profile of the OxlT-antibody complexes in the presence of 10 mM oxalate (OxlT-Fab complex, blue line; OxlT-Fv complex, green line). The same column was equilibrated with a buffer conposed of 20 mM MES-KOH (pH 6.2), 100 mM potassium acetate, 10 mM potassium oxalate, and 0.02% DDM. OxlT (0.16 mg protein) was mixed with the excess antibody fragment (0.32 mg protein), incubated for 1 h on ice, and subjected to the SEC. OxlT alone (0.16 mg protein) was separated on the same column (red line). Peak 6: OxlT-D5901Fab complex; peak 7: OxlT-20D033Fv complex; peak 8: free OxlT; peak 9: free D5901Fab; and peak 10: free 20D033Fv. **d** SDS-PAGE analysis of the corresponding peak fractions in (c). The theoretical molecular masses of the D5901Fab, 20D033Fv and OxlT are 47.2 kDa (Fab_L: 23.6 kDa, Fab_H: 23.6 kDa), 26.3 kDa (Fv_L: 12.0 kDa, Fv_H: 14.3 kDa), and 44.5 kDa, respectively. In panels a-d, the experiments were repeated three times independently. Uncropped versions of the gels in panels b and d are provided in the accompanying Source Data file.

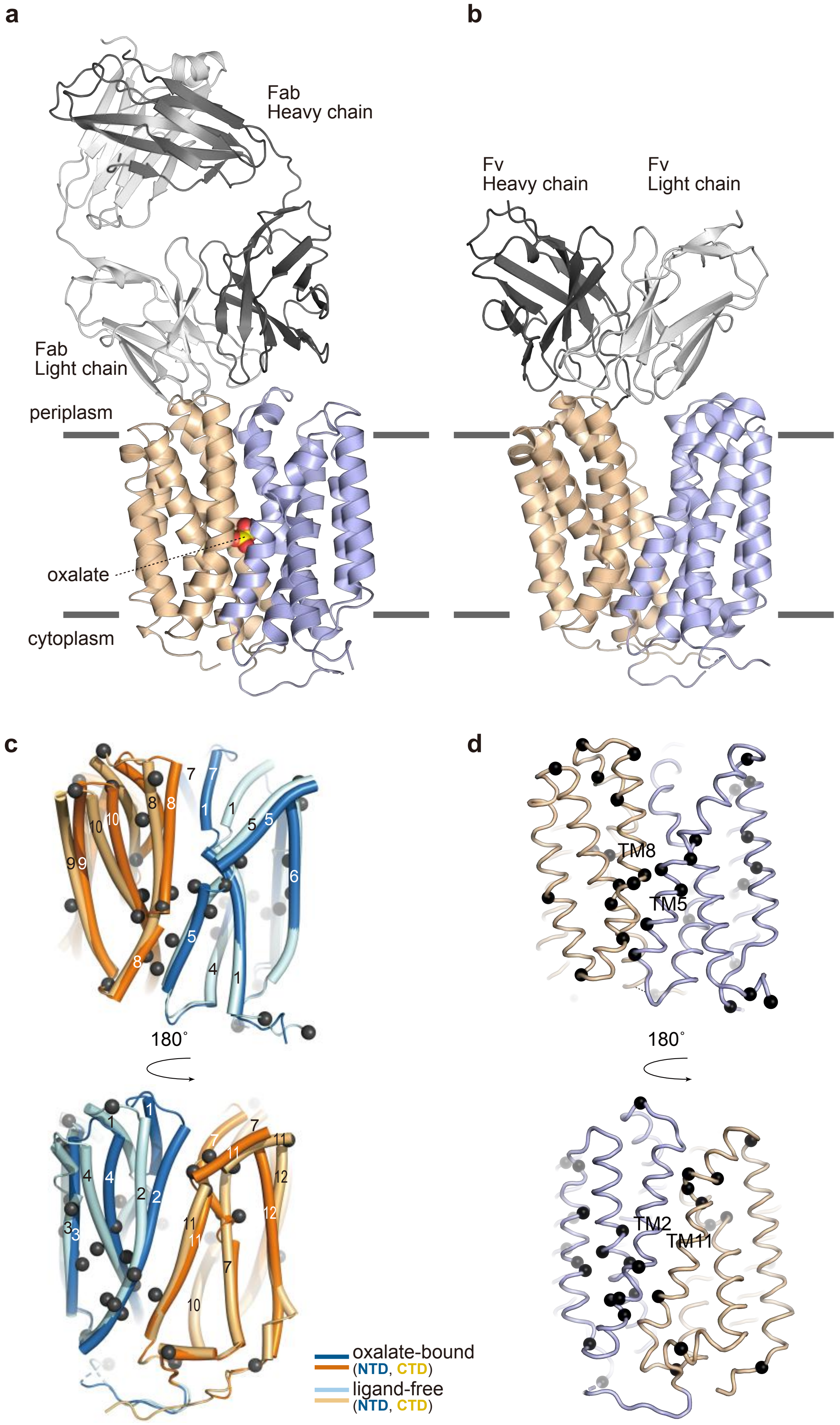

Supplementary Figure 2. **Structure of OxlT. a** The oxalate-bound OxlT in complex with an Fab fragment, D5901Fab (PDB ID 8HPK). **b** the ligand-free OxlT in complex with an Fv fragment, 20D033Fv (PDB ID 8HPJ). **c, d** Positions of the glycine residues mapped on the superposition of oxalate-bound and ligand-free OxlT (c) and the occluded OxlT structure (d), viewed from two different orientations. The black spheres indicate the Cα atoms of glycine residues.

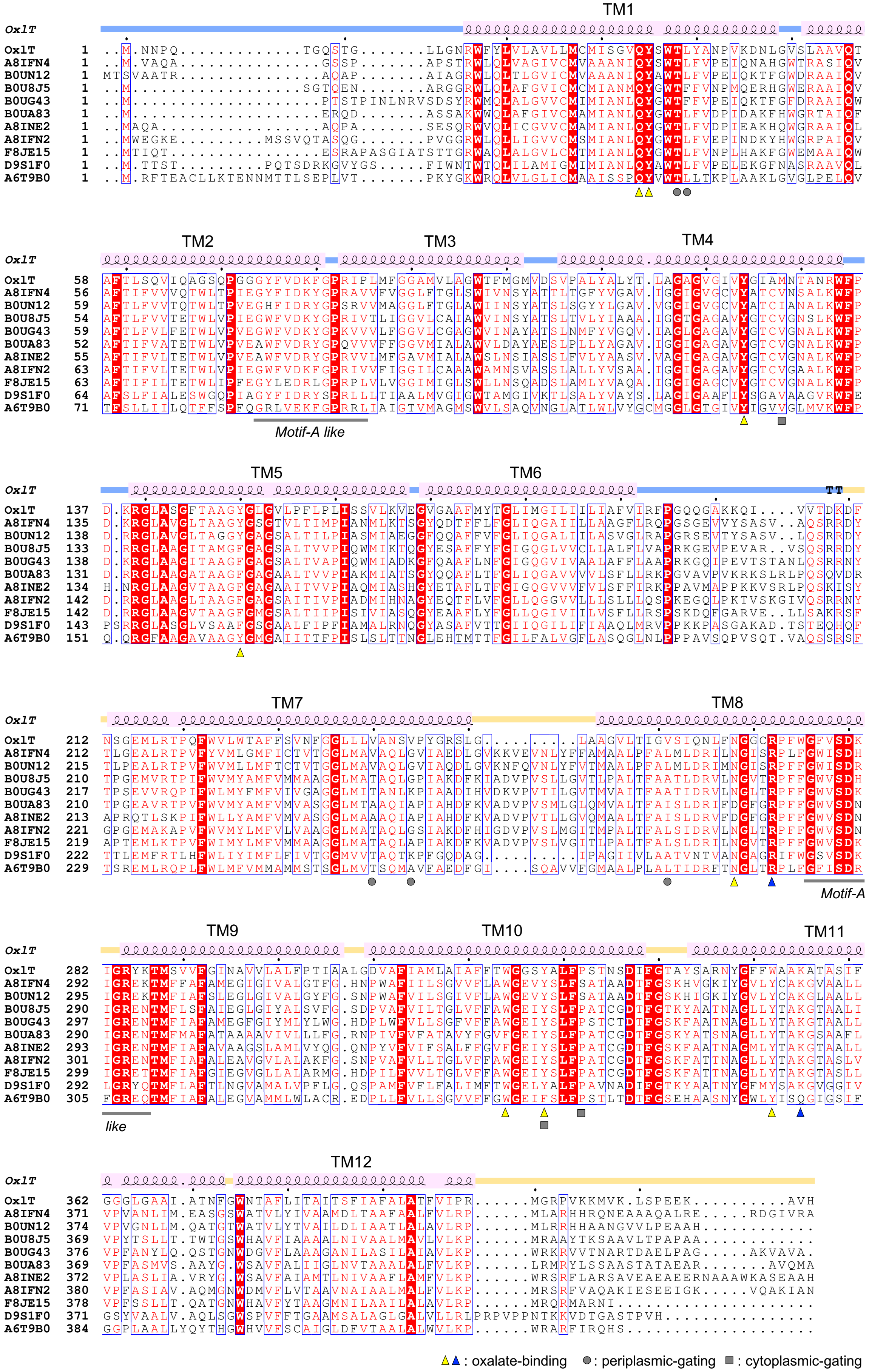

Supplementary Figure 3. **Amino acid sequence alignment of the OFA family proteins in MFS and the secondary structures of OxlT.** Ten entries from oxalate/formate antiporter (InterPro 026355) were aligned via the structure-based sequence using PROMALS3D (Pei *et al. Nucleic Acid Res.* 36, 2295, 2008); the alignment was drawn by the ESPript3.0 server (https://espript.ibcp.fr). Note, all entries except of OxlT are uncharacterised.

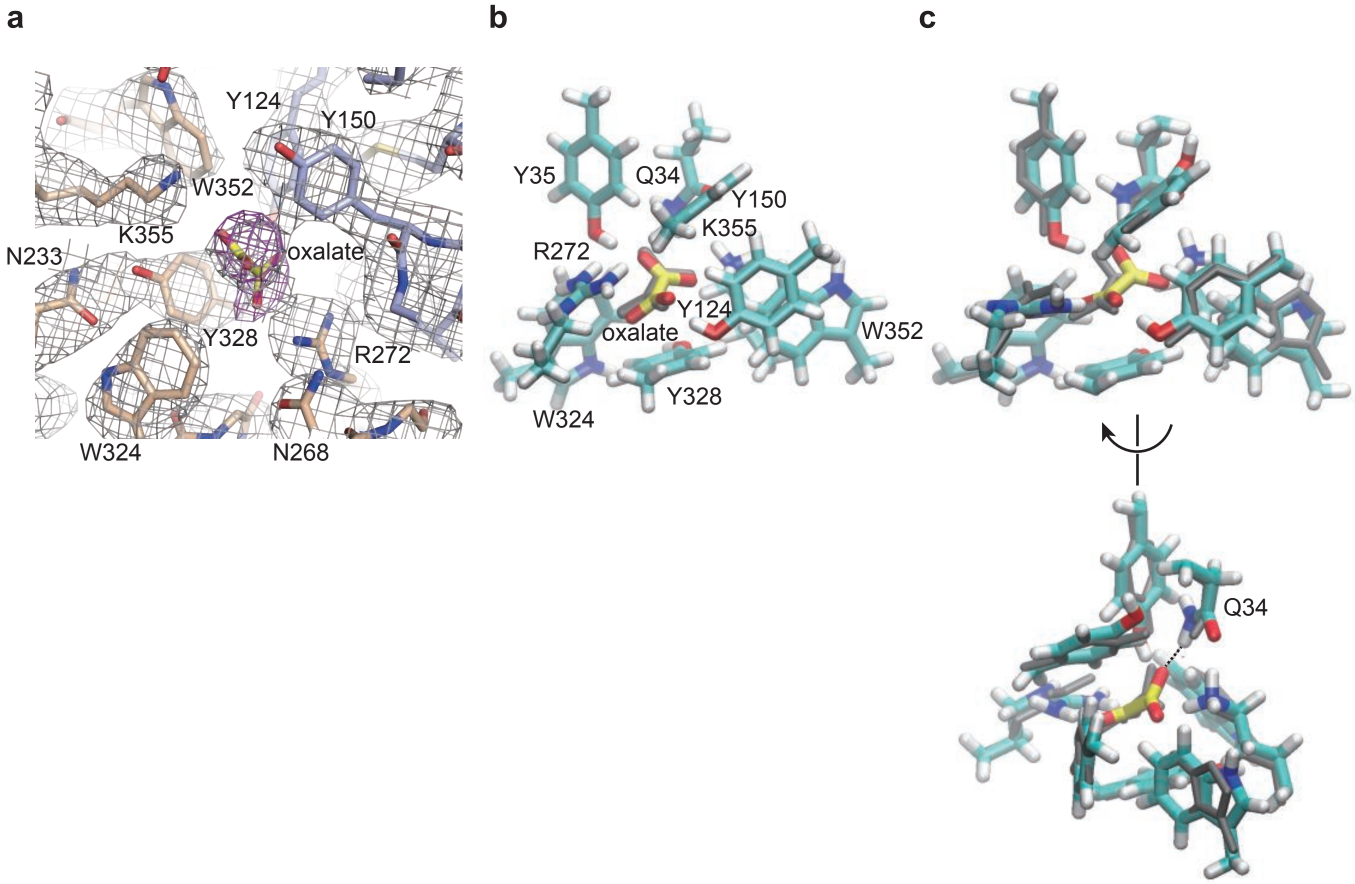

Supplementary Figure 4. **Oxalate binding at the occluded OxlT. a** Simulated-annealing omit map of for the bound oxalate molecule, shown in purple at 3.5 σ. $2F_o$-F_c map at the binding site in grey at 1.5 σ is also shown. **b** The QM-calculated structure of the oxalate binding site at the occluded OxlT. Binding site residues were set frozen, whereas the oxalate molecule was set free. The oxalate ion in the crystal structure is shown in grey. **c** The QM/MM-calculated structure of the oxalate binding site at the occluded OxlT. The QM/MM calculation was performed by applying the oxalate and neighbouring nine residues for QM and the other part of the transporter for MM calculation. Slight rearrangement of the residues in the binding site was observed from the crystal structure shown in grey, such as an additional H-bond formation with Gln34 and the oxalate.

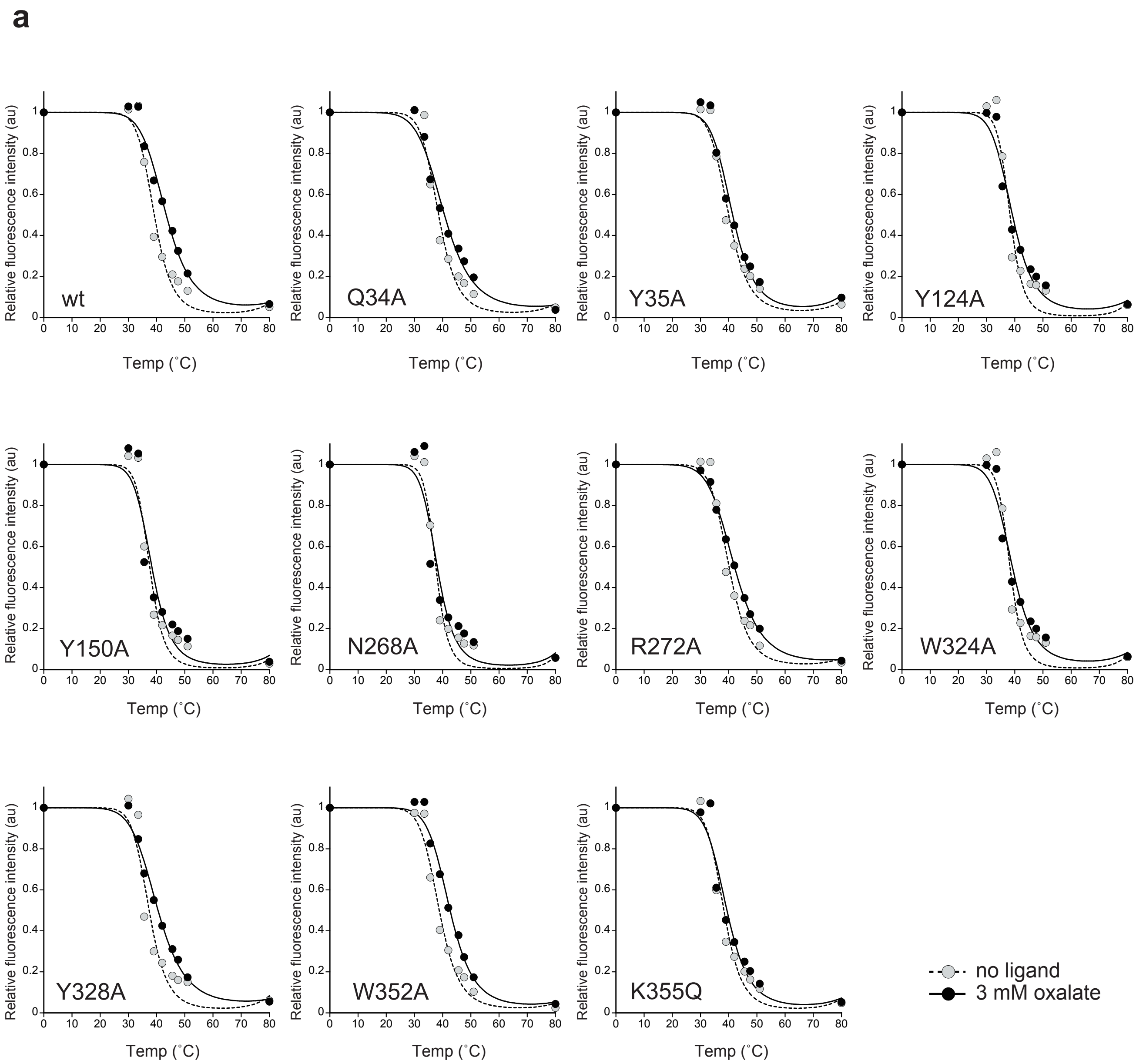

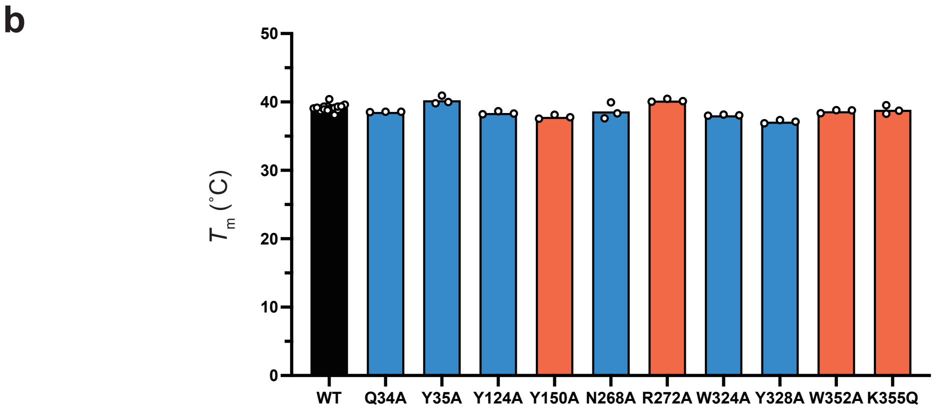

Supplementary Figure 5. **GFP-TS assay of OxlT-GFP fusion proteins. a** Representative thermal melt curves of OxlT-GFP fusion proteins analaysed by GFP-TS. au: arbitrary unit. **b** The apparent melting temperature (T_m) values of wild-type and mutant OxlT in the absence of oxalate. The bars represent the means of three independent experiments.

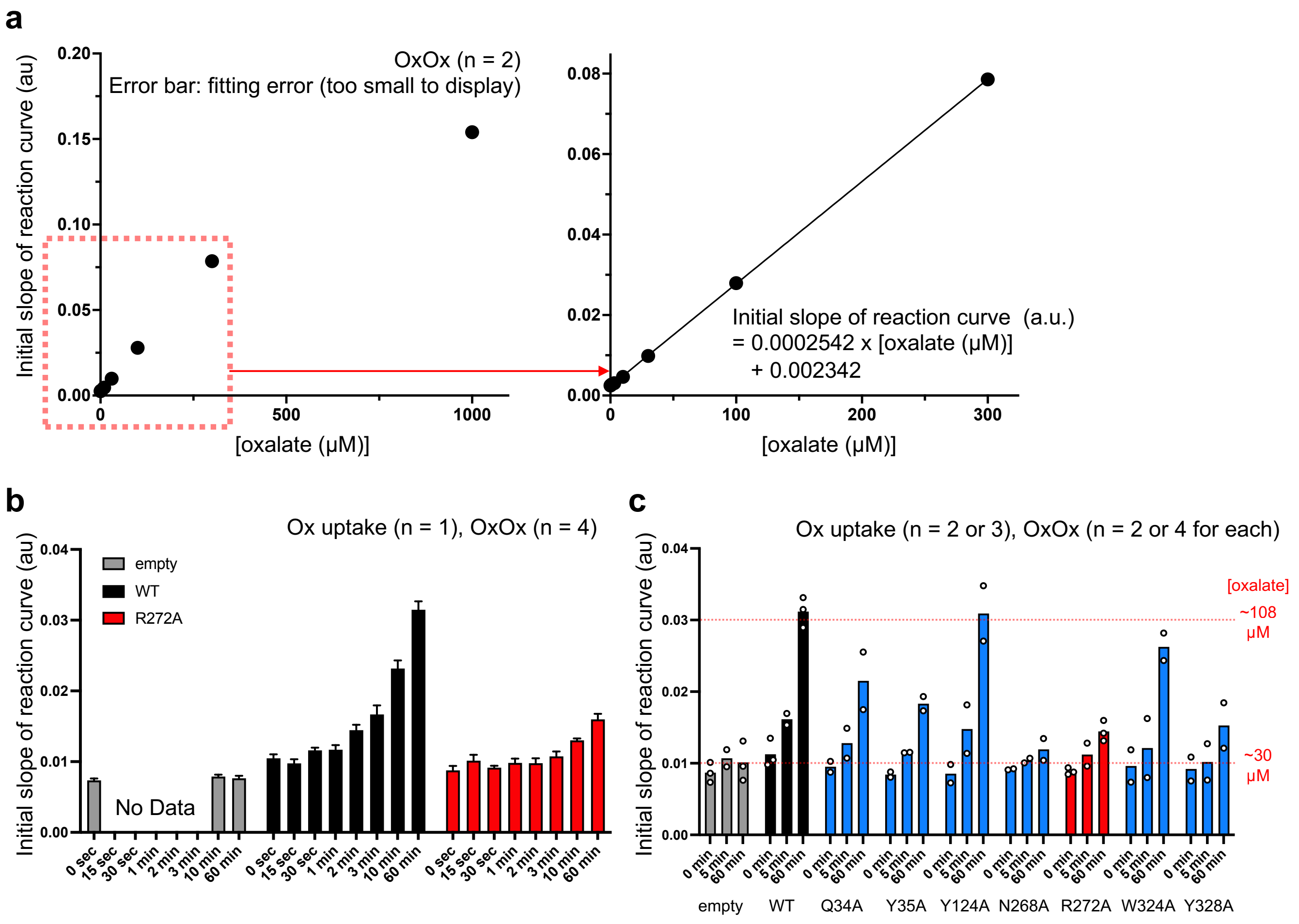

Supplementary Figure 6. **Validation of the OxOx system for proteoliposome oxalate uptake assays.**
a Correlation between the oxalate concentrations and the initial slopes of the fluorometric-time reaction curves in the OxOx assay. Oxalate concentrations are indicated in the figures, and the standard solutions are prepared in 50 mM MOPS-KOH and 10 mM potassium formate, pH 7.0. The OxOx reaction was performed and analyzed as described in the Materials and Methods section, where slope values are linearly correlated between 0 and 300 μM. The experiment was performed once with technical duplicates. **b** Oxalate uptake by the liposomes reconstituted with wild-type or R272A OxlT and without OxlT (empty). The total amount of oxalate inside the (proteo)liposome was measured by the OxOx assay at different time points, as indicated in the figure. Data represent the initial slope values ± fitting errors for their determination by a linear regression analysis. The largest observed value on the wild-type OxlT proteoliposome was ~0.03, which roughly corresponds to 100 μM in the ~50 μL liposome lysate. The experiment was performed once with technical quadruplicates. **c** The non-normalized graph of Fig. 2e in the main text showing that the slope values are within the reliable range for the OxOx assay. The bars represent the means of the results in three (data at 0 min and 60 min for empty, WT, and R272A) or two (others) independent experiments. au: arbitrary unit.

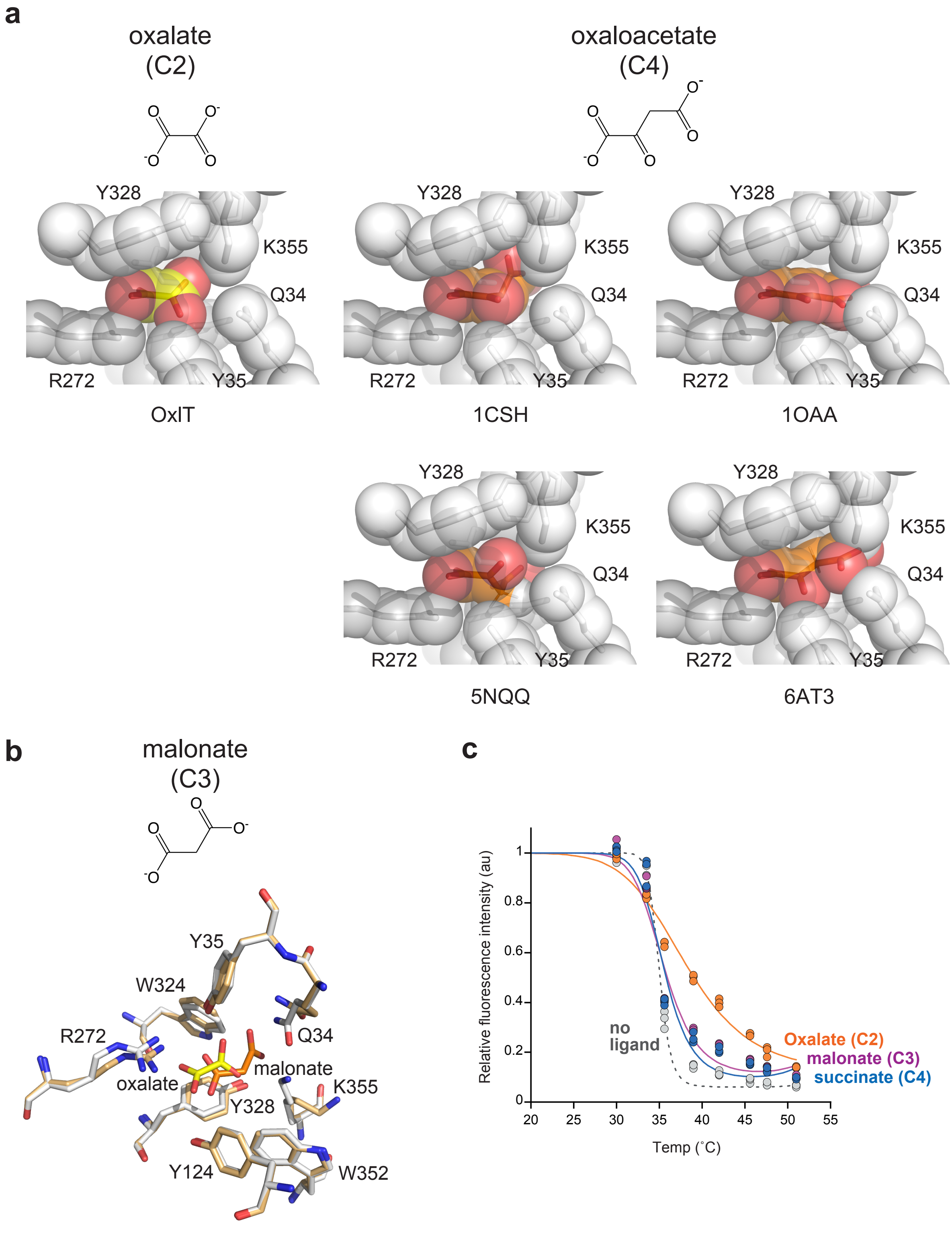

Supplementary Figure 7. **Presumed binding of different dicarboxylates at the substrate-binding site in OxlT.** **a** The models of oxalate, the C2 dicarboxylate, and oxaloacetate, a C4 dicarboxylate intermediate in the Krebs cycle, bound to the occluded OxlT, coloured white (PDB ID 8HPK). The oxaloacetate models with several different representative conformers observed in the PDB were simply supplanted with oxalate in the crystal structure; van der Waals surfaces are shown as spheres. **b** Docking model of the malonate-bound OxlT (OxlT, light orange; malonate, orange), superposed to the crystal structure of the oxalate-bound OxlT (OxlT, white; oxalate, yellow). **c** The thermal melting curves in the absence and presence of 10 mM dicarboxylates. The apparent melting temperature (T_m) values calculated from the curves are as follows: no ligand, 35.0 ± 0.15 ˚C; oxalate (C2 dicarboxylate) added, 38.5 ± 0.48 ˚C; malonate (C3 dicarboxylate) added, 35.7 ± 0.51 ˚C; succinate (C4 dicarboxylate) added, 35.6 ± 0.47 ˚C. The data points are from three independent experiments, and their average values were fitted to the Gibbs–Helmholtz equation to estimate the T_m values. au: arbitrary unit.

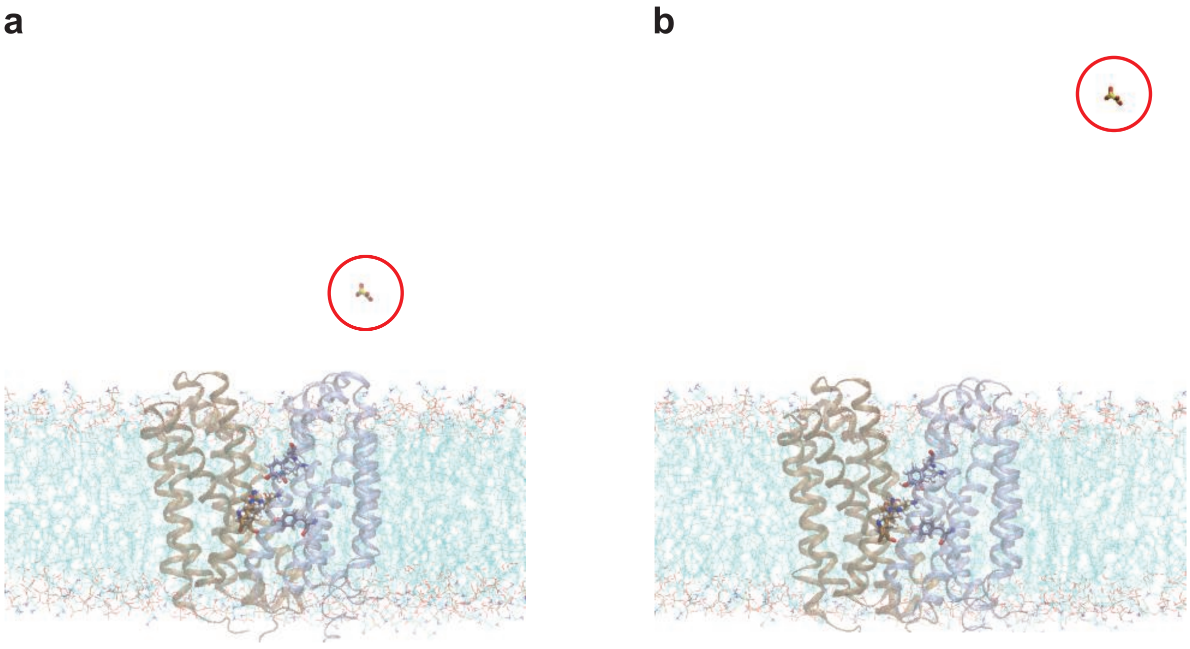

Supplementary Figure 8. **The initial positions of the binding oxalate in simulations initiated from the outward-open conformation (PDB ID 8HPJ), with Lys355 protonated (a) and deprotonated (b).**

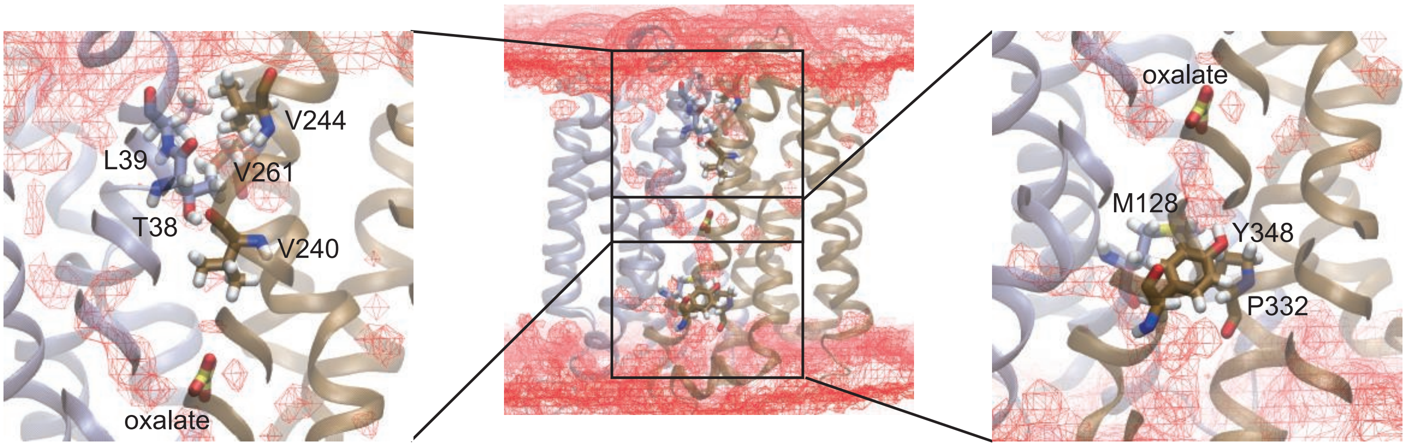

Supplementary Figure 9. **Water density in the occluded state during the simulation.** The iso-surface of the relative density value of 0.5 to the bulk water is shown in red wires.

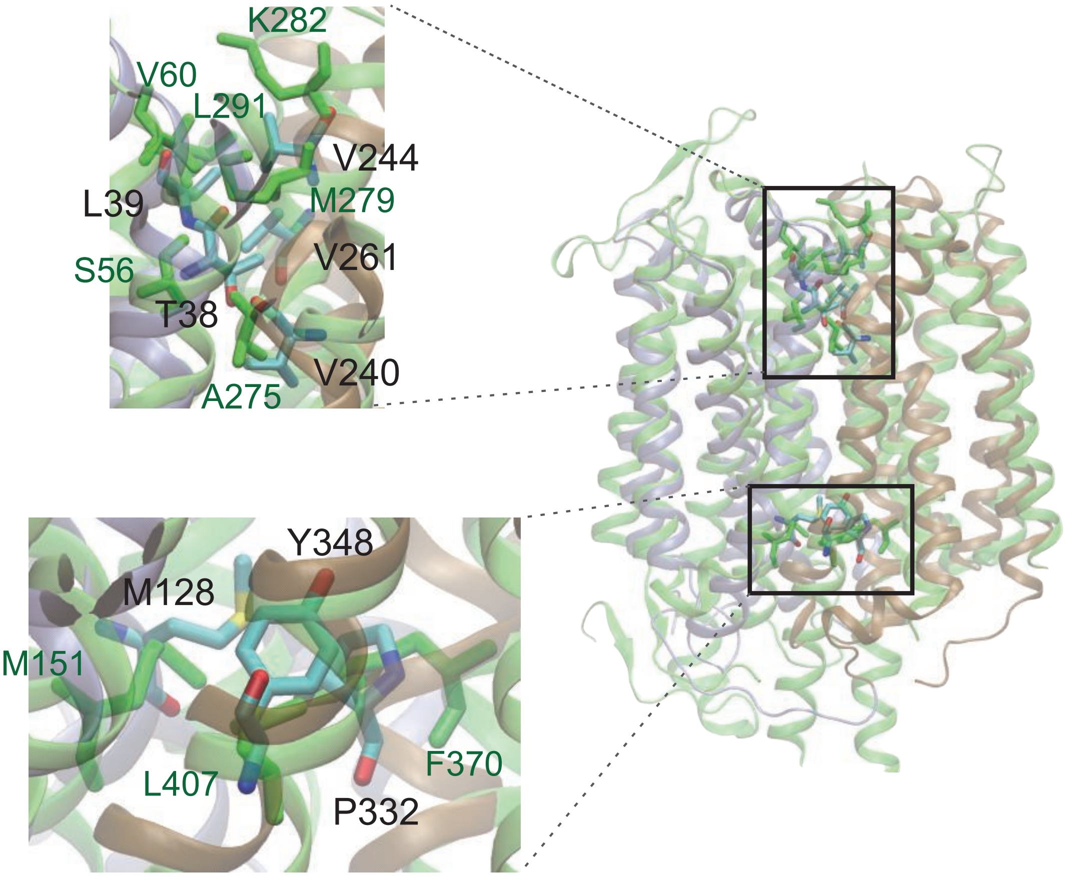

Supplementary Figure 10. **Structural alignment of OxlT (PDB ID 8HPK) and NarK (PDB ID 4U4W).**
The NarK transporter is in green. Residues in OxlT and NarK located at similar positions are labelled in black and green, respectively. The NarK periplasmic gate consists of Ser56 and Val60 in TM1, Ala275 and Met279 in TM7 and Leu291 in TM8, whereas the NarK cytoplasmic gate consists of Met151 in TM4, Phe370 in TM10 and Leu407 in TM11.

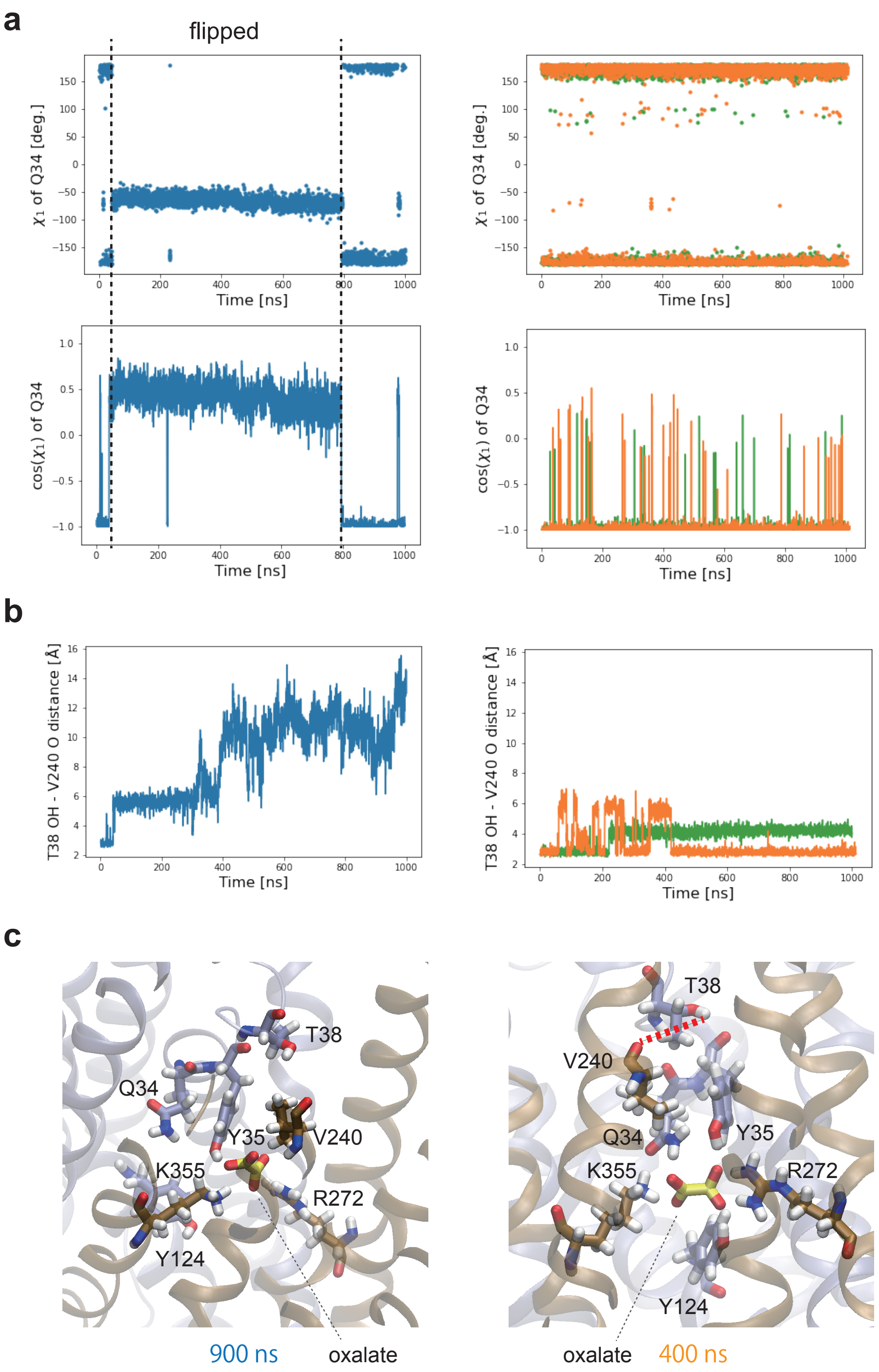

Supplementary Figure 11. **Gln34 side chain and hydrogen bond between Thr38 and Val240 in the simulation from the occluded conformation with oxalate (PDB ID 8HPK). a** The side chain dihedral χ_1 of Gln34 is shown for three independent trajectories with different colours. The flip of Gln34 side chain can be characterised by the change of the side chain dihedral χ. **b** The hydrogen bond donor and acceptor distance between Thr38 and Val240 is shown for three independent trajectories with different colours. **c** Snapshots of the binding site are shown. Data are derived from trajectories shown in Fig. 4d; the results from the trajectory with conformational transition (in blue) and the other two without conformational transition (in orange and green) are shown in the left and right subpanels, respectively.

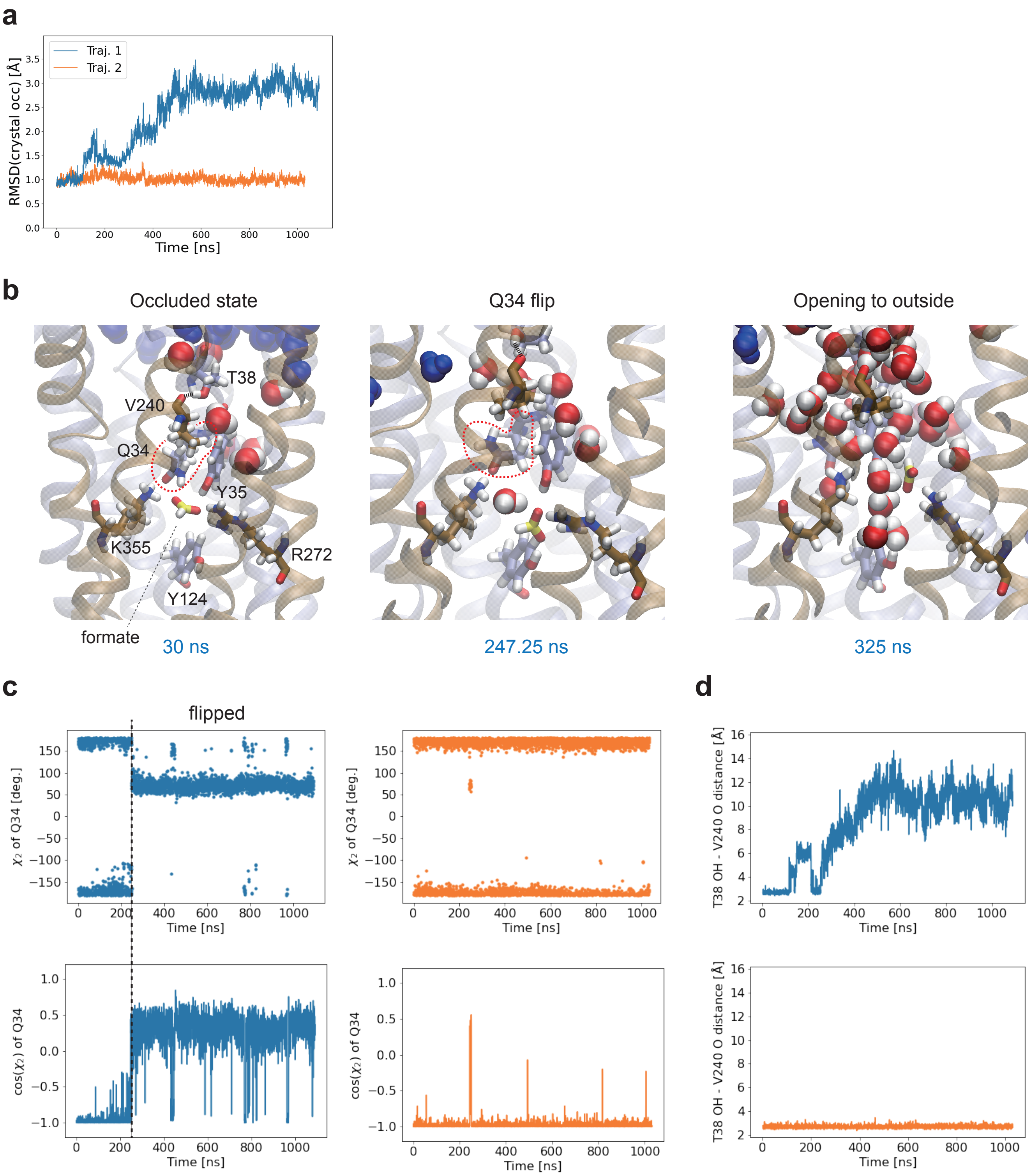

Supplementary Figure 12. **Simulation results from the occluded conformation (PDB ID 8HPK) with formate in the binding site. a** RMSD plot for two independent trajectories. **b** Representative snapshots from a trajectory showing a transition from the occluded to the outward-open conformations. **c** The side chain dihedral χ_2 of Gln34 is shown for two independent trajectories with different colours. Note that χ_1 of Gln34 did not show a significant change upon the Gln34 flip in this case. **d** The hydrogen bond donor and acceptor distance between Thr38 and Val240 is shown for two independent trajectories with different colours.

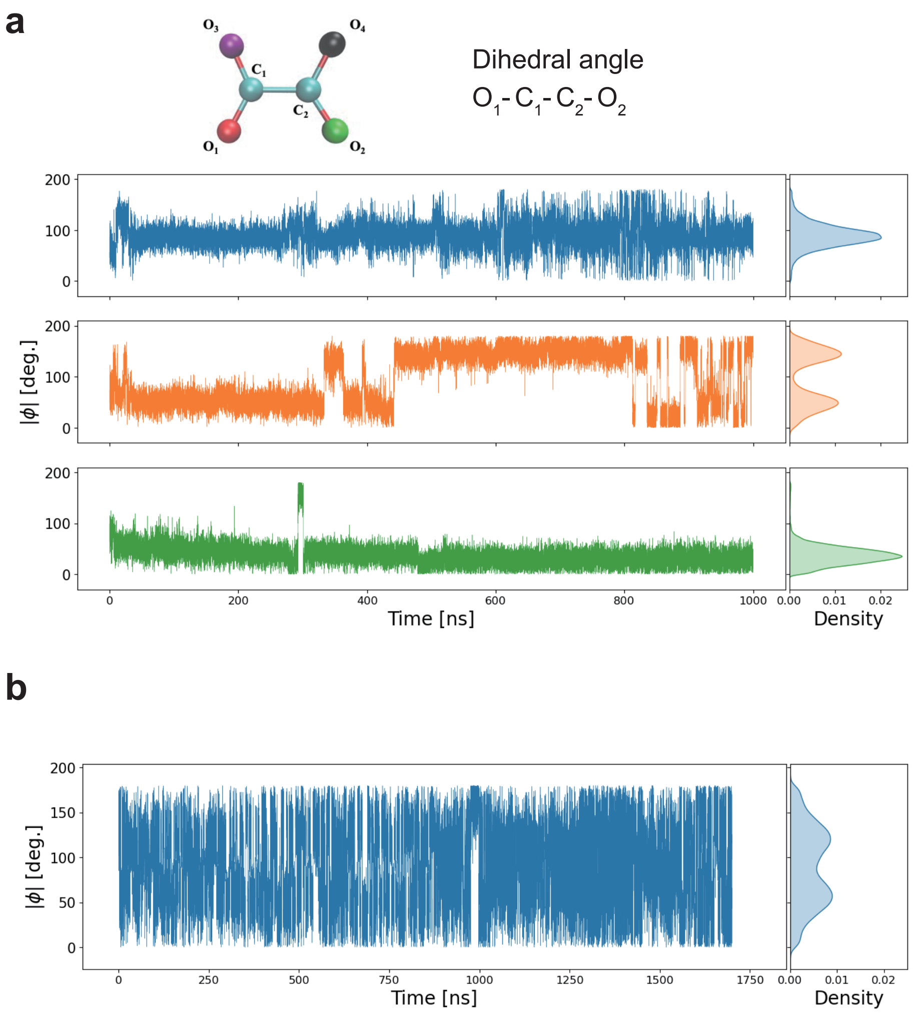

Supplementary Figure 13. **OxlT-bound oxalate conformation during MD simulations. a** The dihedral angle of the oxalate ion in the binding site in MD simulations based on the oxalate-bound occluded OxlT crystal structure (PDB ID 8HPK) is shown for three independent trajectories in the same colour scheme as in Fig. 4d. **b** The dihedral angle of the spontaneously bound oxalate ion in the MD simulation started from the ligand-free outward-facing OxlT crystal structure (PDB ID 8HPJ) with protonated Lys355 is shown.

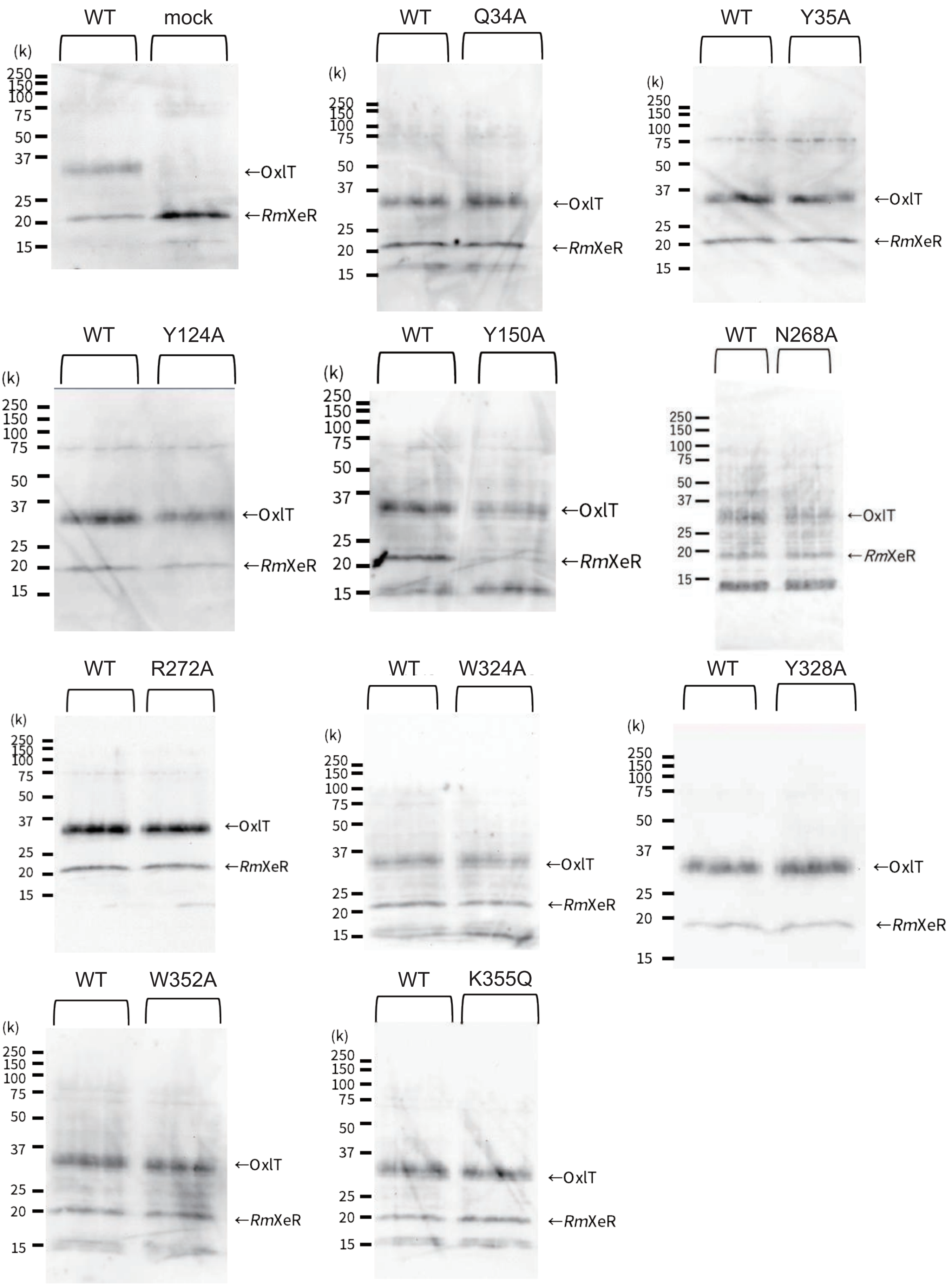

Supplementary Figure 14. **Western blots of samples used for *in cellulo* oxalate transport assay shown in Fig. 2d.** The expression of His-tagged OxlT in recombinant *E. coli* cells was detected by Penta·His Antibody (QIAGEN). Image J software was used to quantify the band intensities (Schneider *et al., Nat Methods,* 9, 671, 2012), and the average values of triplicated samples were used to estimate the relative expression levels. The results of WT vs. mock, WT vs. R272A, and WT vs. K355Q are reposted from ref. 36 (Hayashi *et al., Prot. Sci.*, 30, 2162, 2012) for comparison. Uncropped versions of the blots are provided in the accompanying Source Data file.

Supplementary Table 1. X-ray crystallographic data collection and refinement statistics.

	**OxlT-Fab (oxalate-bound form)** (PDB 8HPK)	**OxlT-Fv (ligand-free form)** (PDB 8HPJ)
**Data collection**		
Space group	$P2_12_12$	$P2_1$
Cell dimensions		
a, b, c (Å)	114.95, 233.19, 50.77	76.87, 181.67, 81.17
α, β, γ (°)	90, 90, 90	90, 111.37, 90
Resolution (Å)	46.64-2.60 (2.94-2.60)[a]	47.26-3.10 (3.40-3.10)
R_{merge}	34.5(454.6)[b]	83.9(332.3)[b]
$I/\sigma(I)$	16.45 (1.71)[b]	5.01 (1.26)[b]
$CC_{1/2}$	99.7 (65.1)[b]	98.0 (68.6)[b]
Ellipsoidal completeness	92.9 (77.4)[b]	88.4 (54.8)[b]
Spherical completeness	52.4(8.8)[b]	69.8(14.4)[b]
Redundancy	99.8 (72.1)[b]	24.8 (24.5)[b]
**Refinement**		
Resolution (Å)	21.08-3.00 (3.11-3.00)	46.23-3.30 (3.42-3.30)
No. reflections	20960	25465
R_{work} / R_{free}	23.7/27.8 (28.5/30.0)	26.0/28.6 (38.9/39.2)
No. atoms		
Protain	6200	9517
Oxalate	6	-
B factors		
Protein	51.2	53.9
Oxalate	42.4	-
R.m.s. deviations		
Bond lengths (Å)	0.003	0.003
Bond angles (°)	0.59	0.61

[a]Values in parentheses are for highest-resolution shell.

[b]Values reported by STARANISO anisotropy & Bayesian estimation server.

Supplementary Table 2. Data statistics per resolution shell after the anisotropy correction.

OxlT-Fab (oxalate-bound form)

Resolution	R_{merge}	$I/\sigma(I)$	$CC_{1/2}$	Ellipsoidal completeness	Spherical completeness.	Redundancy
46.64 - 9.18	11.1	41.81	99.6	99.9	99.9	84.8
9.18 - 7.18	14.3	40.20	99.9	100.0	100.0	97.5
7.18 - 6.24	20.2	32.17	99.9	100.0	100.0	100.6
6.24 - 5.64	23.4	27.25	99.9	100.0	100.0	101.6
5.64 - 5.22	24.6	25.07	99.9	100.0	100.0	103.0
5.22 - 4.90	24.2	24.07	99.9	100.0	100.0	102.8
4.90 - 4.64	27.3	21.80	99.9	100.0	100.0	103.3
4.64 - 4.44	33.3	19.24	99.9	100.0	100.0	103.9
4.44 - 4.26	42.2	16.42	99.9	100.0	100.0	104.4
4.26 - 4.11	52.2	13.82	99.8	100.0	100.0	104.3
4.11 - 3.97	60.2	11.69	99.2	96.1	96.1	105.1
3.97 - 3.85	86.5	10.27	98.7	95.7	91.6	104.7
3.85 - 3.73	91.4	8.84	98.7	96.4	85.5	105.1
3.73 - 3.61	105.0	8.49	99.4	91.5	75.2	103.4
3.61 - 3.49	111.4	7.03	99.2	90.6	69.1	103.6
3.49 - 3.38	153.1	5.86	97.9	89.5	59.9	102.1
3.38 - 3.26	169.9	5.12	96.9	86.7	51.9	101.9
3.26 - 3.12	197.9	4.46	94.6	74.4	36.4	98.4
3.12 - 2.94	247.2	3.61	88.2	76.2	23.2	94.4
2.94 - 2.60	454.6	1.71	65.1	77.4	8.8	72.1

OxlT-Fv (ligand-free form)

Resolution	R_{merge}	$I/\sigma(I)$	$CC_{1/2}$	Ellipsoidal completeness	Spherical completeness.	Redundancy
47.26 - 9.54	23.3	12.18	98.7	99.8	99.8	24.5
9.54 - 7.56	27.9	10.79	98.8	100.0	100.0	24.8
7.56 - 6.60	36.5	9.02	98.9	100.0	100.0	25.3
6.60 - 6.00	43.8	8.07	98.2	100.0	100.0	25.6
6.00 - 5.57	53.6	6.91	97.9	100.0	100.0	25.5
5.57 - 5.23	57.5	6.67	96.8	100.0	100.0	24.8
5.23 - 4.97	59.8	6.70	97.0	100.0	100.0	25.3
4.97 - 4.75	62.7	6.60	96.7	99.9	99.9	25.5
4.75 - 4.57	97.0	5.13	95.3	99.6	99.6	24.6
4.57 - 4.30	173.2	2.29	61.9	56.8	56.8	16.4
4.30 - 4.18	135.8	3.45	92.9	99.2	99.2	24.7
4.18 - 4.06	109.3	4.33	91.8	99.4	99.4	25.0
4.06 - 3.96	177.4	2.76	86.6	99.5	99.5	25.0
3.96 - 3.87	150.6	3.46	86.2	97.8	97.8	25.6
3.87 - 3.78	171.1	3.08	85.4	95.8	95.8	25.6
3.78 - 3.70	269.4	1.97	81.3	92.1	92.1	26.3
3.70 - 3.62	281.0	1.95	80.6	87.5	87.3	25.7
3.62 - 3.53	261.9	1.82	82.5	79.8	72.8	25.7
3.53 - 3.40	265.9	1.87	71.3	64.3	43.9	25.3
3.40 - 3.10	332.3	1.26	68.6	54.8	14.4	24.5

Supplementary Table 3. Oxalate dihedral angle and binding distances derived from QM and QM/MM geometry optimisations.

	Dihedral angle	Binding distance				
	O_1-C_1-C_2-O_2 angle (°)	O_1-Y124(OH) (Å)	O_2-K355(NZ) (Å)	O_3-R272(NH2) (Å)	O_4-Y35(OH) (Å)	O_4-Q34(NE2) (Å)
Crystal structure (PDB ID 8HPK)	60.1	3.0	3.2	2.6	2.3	> 3
QM<br>9 frozen residues (Q34, Y35, Y124, Y150, R272, W324, Y328, W352, K355) + free oxalate						
B3LYP	68.2	2.7	2.8	2.8	2.6	> 3
B3LYP-D3BJ	68.2	2.7	2.8	2.8	2.6	> 3
QM/MM<br>Oxalate, Q34, Y35, Y124, R272, K355 (QM) + other region of the protein (MM)						
B3LYP-D3BJ	50.2	2.7	2.6	2.6	2.8	2.8
QM/MM<br>Oxalate, Q34, Y35, Y124, Y150, R272, W324, Y328, W352, K355 (QM) + other region of the protein (MM)						
B3LYP-D3BJ	52.3	2.7	2.7	2.6	2.7	2.8

The dihedral angle and binding distances of oxalate in the OxlT binding site are shown for the crystal structure and the optimized geometries from QM and QM/MM calculations. The results of the QM calculation with the nine frozen residues and free oxalate using either the B3LYP or B3LYP-D3BJ functionals are displayed. The results of the QM/MM calculations using the B3LYP-D3BJ functional with two different definitions of the QM region are also presented.

Supplementary Table 4. Relative binding free energy of oxalate to OxlT calculated with the molecular mechanics / generalized Born surface area (MM/GBSA) method.

Stage	*Average*	*Std. Dev.*	*Std. Err. of Mean*
OxlT-op-dih90	-13.5	7.2	0.3
OxlT-occ-dih90	-35.5	6.3	0.4
OxlT-occ-dih50	-36.6	4.5	0.7

It was determined for three stages: the outward-open conformation (PDB ID 8HPJ) with the oxalate dihedral ~ 90 degree (OxlT-op-dih90), the occluded conformation (PDB ID 8HPK) with the oxalate dihedral ~ 90 degree (OxlT-occ-dih90), and the occluded conformation with the oxalate dihedral ~ 50 degree (OxlT-occ-dih50). The values are shown in kcal/mol unit.

Supplementary Table 5. Summary of simulation systems.

	Initial structure	*Lys355 protonation*	*Bound ligand*	*Number of Trajectories*	*Total Simulation Time [ns]*
System1	Outward-open (PDB ID 8HPJ)	Protonated	N/A	1	1700
System2	Outward-open (PDB ID 8HPJ)	Deprotonated	N/A	1	1700
System3	Occluded (PDB ID 8HPK)	Protonated	Oxalate	3	3000
System4	Occluded (PDB ID 8HPK)	Protonated	Formate	2	2000

Supplementary Table 6. Results of the Dunnett's multiple comparisons tests for Figure 2c and 2e.

	Mean Diff.	95.00% CI of diff.	Adjusted *P* Value
Figure 2c			
WT vs. Q34A	2.399	1.533 to 3.264	<0.0001
WT vs. Y35A	2.760	1.894 to 3.625	<0.0001
WT vs. Y124A	3.755	2.889 to 4.621	<0.0001
WT vs. Y150A	3.306	2.440 to 4.171	<0.0001
WT vs. N268A	3.713	2.847 to 4.579	<0.0001
WT vs. R272A	2.519	1.653 to 3.385	<0.0001
WT vs. W324A	3.818	2.952 to 4.684	<0.0001
WT vs. Y328A	0.9026	0.03713 to 1.768	0.0363
WT vs. W352A	0.6586	-0.2069 to 1.524	0.2442
WT vs. K355Q	3.234	2.368 to 4.099	<0.0001
Figure 2e[a]			
WT vs. empty	67.60	41.16 to 94.04	<0.0001
WT vs. Q34A	31.00	1.437 to 60.56	0.0384
WT vs. Y35A	41.30	11.74 to 70.86	0.0058
WT vs. Y124A	0.8500	-28.71 to 30.41	0.9999
WT vs. N268A	61.70	32.14 to 91.26	0.0002
WT vs. R272A	53.67	27.22 to 80.11	0.0003
WT vs. W324A	15.75	-13.81 to 45.31	0.4932
WT vs. Y328A	51.00	21.44 to 80.56	0.0010

[a]The data at 60 min were analysed.
