# Supplementary material for: Structure and mechanism of oxalate transporter OxlT in an oxalate-degrading bacterium in the gut microbiota
Source: Nat Commun. 2023 Apr 3;14:1730. doi: 10.1038/s41467-023-36883-5 (PMC10070484; doi:10.1038/s41467-023-36883-5)
Supplement: Supplementary file 3 — Description of Additional Supplementary Files [file 41467_2023_36883_MOESM3_ESM.pdf]

<u>Description of Additional Supplementary Files</u>

Title: Supplementary Data 1

Description: Sequences of oligonucleotides used for mutation in the expression vectors of OxlT.

Title: Supplementary Movie 1

Description: The first 600 ns trajectory of OxlT starting from the occluded conformation, which remained in the occluded state, is shown. The representation is the same as Fig. 4e, except all water molecules are shown in the CPK color.

Title: Supplementary Movie 2

Description: The first 500 ns trajectory of OxlT starting from the occluded conformation with a transition to the outward-open state is shown. The representation is the same as Fig. 4f, except all water molecules are shown in the CPK color.
