# Supplementary material for: Structure and mechanism of oxalate transporter OxlT in an oxalate-degrading bacterium in the gut microbiota
Source: Nat Commun. 2023 Apr 3;14:1730. doi: 10.1038/s41467-023-36883-5 (PMC10070484; doi:10.1038/s41467-023-36883-5)
Supplement: Supplementary file 7 — Reporting Summary [file 41467_2023_36883_MOESM7_ESM.pdf]

nature portfolio

Corresponding author(s): Tatsuro shimamura, Kei-ichi Okazaki, Teruhisa Hirai, Atsuko Yamashita

Last updated by author(s): Feb 12, 2023

Reporting Summary

Nature Portfolio wishes to improve the reproducibility of the work that we publish. This form provides structure for consistency and transparency in reporting. For further information on Nature Portfolio policies, see our Editorial Policies and the Editorial Policy Checklist.

Statistics

For all statistical analyses, confirm that the following items are present in the figure legend, table legend, main text, or Methods section.

n/a	Confirmed	
☐	☒	The exact sample size (n) for each experimental group/condition, given as a discrete number and unit of measurement
☐	☒	A statement on whether measurements were taken from distinct samples or whether the same sample was measured repeatedly
☐	☒	The statistical test(s) used AND whether they are one- or two-sided<br>*Only common tests should be described solely by name; describe more complex techniques in the Methods section.*
☒	☐	A description of all covariates tested
☒	☐	A description of any assumptions or corrections, such as tests of normality and adjustment for multiple comparisons
☐	☒	A full description of the statistical parameters including central tendency (e.g. means) or other basic estimates (e.g. regression coefficient) AND variation (e.g. standard deviation) or associated estimates of uncertainty (e.g. confidence intervals)
☐	☒	For null hypothesis testing, the test statistic (e.g. F, t, r) with confidence intervals, effect sizes, degrees of freedom and P value noted<br>*Give P values as exact values whenever suitable.*
☒	☐	For Bayesian analysis, information on the choice of priors and Markov chain Monte Carlo settings
☒	☐	For hierarchical and complex designs, identification of the appropriate level for tests and full reporting of outcomes
☒	☐	Estimates of effect sizes (e.g. Cohen's d, Pearson's r), indicating how they were calculated

Our web collection on statistics for biologists contains articles on many of the points above.

Software and code

Policy information about availability of computer code

Data collection	BSS (Beamline Scheduling Software, SPring-8)
Data analysis	KAMO system (BLEND, XDS Version Mar 15 2019, XSCALE), PHASER-2.3.0, 2.5.6, COOT-0.8.1, 0.8.9, Phenix-1.19.2, Refmac-5.8.0069, MOLPROBITY (in Phenix-1.19.2), Pymol-2.5.0, ImageJ-1.53a, MODELLER-9.23, PROPKA 3.1, CHARMM-GUI , AmberTools17, NAMD 2.12, MDAnalysis, Gaussian 16, MMPBSA.py, Antechamber (in AmberTools17), Prism ver. 8, Kaleidagraph ver. 5.0

For manuscripts utilizing custom algorithms or software that are central to the research but not yet described in published literature, software must be made available to editors and reviewers. We strongly encourage code deposition in a community repository (e.g. GitHub). See the Nature Portfolio guidelines for submitting code & software for further information.

Data

Policy information about availability of data

All manuscripts must include a data availability statement. This statement should provide the following information, where applicable:

- Accession codes, unique identifiers, or web links for publicly available datasets
- A description of any restrictions on data availability
- For clinical datasets or third party data, please ensure that the statement adheres to our policy

Coordinates and structure factors for OxlT have been deposited in the Protein Data Bank under the accession numbers 8HPK [https://doi.org/10.2210/pdb8HPK/pdb] (OxlT-fab complex; oxalate-bound occluded form) and 8HPJ [https://doi.org/10.2210/pdb8HPJ/pdb] (OxlT-Fv complex; ligand-free outward-facing form). The MD-related data have been deposited in the Zenodo repository [https://doi.org/10.5281/zenodo.7597686]. Coordinates with the PDB IDs 1PW4 [https://

doi.org/10.2210/pdb1PW4/pdb], 1XF4 [https://doi.org/10.2210/pdb1XF4/pdb], 5B3N [https://doi.org/10.2210/pdb5B3N/pdb], 4U4W [https://doi.org/10.2210/pdb4U4W/pdb], 4U4T [https://doi.org/10.2210/pdb4U4T/pdb], and amino acid sequences of OFA family (IPR026355 [https://www.ebi.ac.uk/interpro/entry/InterPro/IPR026355/]) were used in this study. Source data are provided with this paper.

Human research participants

Policy information about studies involving human research participants and Sex and Gender in Research.

Reporting on sex and gender	No human research is reported in this study.
Population characteristics	No human research is reported in this study.
Recruitment	No human research is reported in this study.
Ethics oversight	No human research is reported in this study.

Note that full information on the approval of the study protocol must also be provided in the manuscript.

Field-specific reporting

Please select the one below that is the best fit for your research. If you are not sure, read the appropriate sections before making your selection.

[x] Life sciences [ ] Behavioural & social sciences [ ] Ecological, evolutionary & environmental sciences

For a reference copy of the document with all sections, see nature.com/documents/nr-reporting-summary-flat.pdf

Life sciences study design

All studies must disclose on these points even when the disclosure is negative.

Sample size	For the GFP-TS assays, transport assays, and the SEC analysis, the number of replication was chosen based on the authors' preliminary experiments or previous experiences (such as described in Hayashi et al. Protein Sci 30, 2161-2169, 2021 or Supplementary Fig. 6). No other relevant life science experiment was reported in this work.
Data exclusions	For the GFP-TS assay (Fig. 2c), the results from the experiments where the incomplete heat denature at 80 C was observed, i.e. the residual fluorescence intensity at 80 C was > 10% of the value from the original sample without heat denature, were excluded, because it is a signature of the inappropriate setting of the experimental condition, such as a wrong ligand concentration, mis-setting of the heat denature temperatures, contamination, etc.. For the functional assay of the transporter using E. coli, experimental sets with low wild-type activities measured on the same day of the experiment were excluded, because it is an indication of the inappropriate setting of experimental conditions, such as a wrong ligand concentration, misalignment of the optics, etc. In addition, a mutant showing low expression levels for the probe proton pump (Y150A) was excluded as described in the manuscript, because such situation doesn't ensure the coupling between transporter activities and probe proton pump activities. Other than these situations, all the measured data were included in the manuscript.
Replication	For the GFP-TS assays (Fig. 2c and Supplementary Fig. 5, 7c) and the SEC analysis (Supplementary Fig. 1), the results from three independent experiments were reported. For the functional assay of the transporter using E. coli (Fig. 2d), all the measurements were performed with technical duplication from a single experiment for each mutant. For the proteoliposome assay (Fig. 2e and Supplementary Fig. 6c), the results from two or three independent experiments were reported. All attempts at replication were successful. No other relevant life science experiment was reported in this work.
Randomization	This study did not include experiments with experimental group allocation and thus no randomization was applied.
Blinding	This study did not include experiments with experimental group allocation and thus no blinding was applied. All experimental samples were prepared and analyzed as described in the manuscript.

Reporting for specific materials, systems and methods

We require information from authors about some types of materials, experimental systems and methods used in many studies. Here, indicate whether each material, system or method listed is relevant to your study. If you are not sure if a list item applies to your research, read the appropriate section before selecting a response.

Materials & experimental systems

n/a	Involved in the study	
[ ]	[x]	Antibodies
[x]	[ ]	Eukaryotic cell lines
[x]	[ ]	Palaeontology and archaeology
[ ]	[x]	Animals and other organisms
[x]	[ ]	Clinical data
[x]	[ ]	Dual use research of concern

Methods

n/a	Involved in the study	
[x]	[ ]	ChIP-seq
[x]	[ ]	Flow cytometry
[x]	[ ]	MRI-based neuroimaging

Antibodies

Antibodies used	D5901Fab (prepared in this study), 20D033Fv (prepared in this study), Penta·His HRP Conjugate (QIAGEN, Cat. No.: 34460, 1:2000 dilution)
Validation	D5901Fab and 20D033Fy are antibody fragments specific for the oxalate transporter OxlT. The antibodies and their fragments were prepared and characterized as described in the manuscript.<br>Penta·His HRP Conjugate was validated by the manufacturer. The information for the conjugated antibody is available at [https://www.qiagen.com/br/products/discovery-and-translational-research/protein-purification/tagged-protein-expression-purification-detection/anti-his-hrp-conjugate-kits], while that for the unconjugated version is available at [https://www.qiagen.com/us/products/discovery-and-translational-research/protein-purification/tagged-protein-expression-purification-detection/anti-his-antibodies-bsa-free?catno=34660]. In addition, low signals for E. coli lysates without transformation of the<br>expression vector for the target protein and signals for a His-tagged purified protein (XeR) were confirmed in our hands, as shown in Supplementary Fig. 14 and the relevant uncropped blots in the Source Data File (loss of the signals for OxlT-derived band for mock-transformed E. coli and detection of the signals for the bands derived from purified His-tagged XeR).

Animals and other research organisms

Policy information about studies involving animals; ARRIVE guidelines recommended for reporting animal research, and Sex and Gender in Research

Laboratory animals	BALB/c mice (7-week-old females) were used for immunization and antibody production.
Wild animals	No wild animals were used in the study.
Reporting on sex	Sex of animals does not influence of the results (i.e. qualities of produced antibodies suitable for crystallization) in this study. Therefore, sex-based analysis is not relevant to this study.
Field-collected samples	No field collected samples were used in the study.
Ethics oversight	All animal experiments conformed to the guidelines of the Guide for the Care and Use of Laboratory Animals of Japan and were approved by the Animal Experimentation Committee at the University of Tokyo (permission number: RAC07101).

Note that full information on the approval of the study protocol must also be provided in the manuscript.
